# Biosensors for the Multiplex Detection of Inflammatory Disease Biomarkers

**DOI:** 10.3390/bios11010011

**Published:** 2020-12-28

**Authors:** Ali Mobasheri

**Affiliations:** 1 Research Unit of Medical Imaging, Physics and Technology, Faculty of Medicine, University of Oulu, FI-90014 Oulu, Finland; ali.mobasheri@oulu.fi; 2 Department of Regenerative Medicine, State Research Institute Centre for Innovative Medicine, LT-08406 Vilnius, Lithuania; ali.mobasheri@imcentras.lt; 3 University Medical Center Utrecht, Departments of Orthopedics, Rheumatology and Clinical Immunology, 3508 GA Utrecht, The Netherlands; a.mobasheri@umcutrecht.nl

A biosensor is an analytical device used for the real-time detection and measurement of a chemical or biochemical substance [1]. It is defined as “an analytical device incorporating a biological material (e.g., tissue, microorganisms, antibodies, natural products, cell receptors, enzymes, nucleic acids, etc.) that combines a biological component with a physicochemical detector. Biosensors are bioanalytical devices that incorporate a biological material, a biologically derived material or a biomimetic material as the recognition molecules, which are either intimately associated with or integrated within a physicochemical transducer or transducing microsystem [2,3,4]. The first biosensor was introduced in the early 1960s by Clark and Lyons. A biosensor can detect an analyte in a sample and estimate its concentration as an electrical signal via a suitable combination of a biological recognition system and an electrochemical transducer [5]. The incorporation of the enzyme glucose oxidase in an electrochemical sensor for detection and measurement of glucose in blood plasma revolutionized the way diabetes is diagnosed and monitored [6]. From humble beginnings that included simple enzyme electrodes, the field of biosensors has steadily progressed with increasing levels of sophistication, leading to the development of immunosensors, optides, optical affinity sensors, lectin-based sensors, and organ on chip-based technologies and nanobiosensors [7,8,9,10,11]. Biosensors have important applications in medical diagnostics, personalized medicine, the food industry, and substance testing laboratories. Biosensor devices are becoming increasingly advanced, sophisticated, inexpensive, and miniaturized [12,13,14]. However, there is an acute need for further innovation in this area, particularly in the field of immunoassays for the multiplex detection of inflammatory disease biomarkers. Portable immunoassays provide the means for rapid testing, but they do not provide the precision, accuracy, and detection limits available with laboratory chemical analytical techniques. Additionally, immunoassays can produce false-positive or false-negative results, depending on the sample matrix. The interpretation of data from immunoassays is challenging, and the spectrum of drugs and biochemicals detected using immunoassays is relatively narrow. We need biosensor platforms that can measure multiple biomarker targets sensitively and accurately, with quantitative readouts, high sensitivity and specificity. In the field of medical diagnostics, we need biosensors capable of multiplex detection of biomarkers of inflammatory diseases. The remit of Biosensors (MDPI) is to provide a dedicated platform and an advanced forum for studies related to the science and technology of biosensors and biosensing. The journal publishes original research articles, comprehensive reviews, and short communications. This Special Issue of Biosensors is entitled: "Biosensors for Multiplex Detection of Biomarkers of Inflammatory Disease". It is dedicated to the topic of biosensors for the multiplex detection of biomarkers of inflammatory diseases. These may include any biomarker that is detectable in a biological sample or body fluid in a clinical setting. Papers may include in vitro studies and studies on preclinical animals, translational models, and human patients. Innovative and original studies on biosensors for the detection and quantification of any cell- or tissue-derived biomarker will be considered. We also welcome studies on biosensors of viral disease, especially papers that advance COVID-19 diagnostics.
